# Bridging Neural and Computational Viewpoints on Perceptual Decision-Making

**DOI:** 10.1016/j.tins.2018.06.005

**Published:** 2018-11

**Authors:** Redmond G. O’Connell, Michael N. Shadlen, KongFatt Wong-Lin, Simon P. Kelly

**Affiliations:** 1 Trinity College Institute of Neuroscience and School of Psychology, Trinity College Dublin, Ireland; 2 Howard Hughes Medical Institute and Department of Neuroscience, Columbia University, New York, NY 10032, USA; 3 Zuckerman Mind Brain Behaviour Institute and Kavli Institute for Brain Science, Columbia University, New York, NY 10032, USA; 4 Intelligent Systems Research Centre, University of Ulster, Magee Campus, Northland Road, Derry, BT48 7JL, UK; 5 School of Electrical and Electronic Engineering, University College Dublin, Dublin, Ireland

**Keywords:** perceptual decision-making, computational modelling, sequential sampling, lateral intraparietal area (LIP)

## Abstract

Sequential sampling models have provided a dominant theoretical framework guiding computational and neurophysiological investigations of perceptual decision-making. While these models share the basic principle that decisions are formed by accumulating sensory evidence to a bound, they come in many forms that can make similar predictions of choice behaviour despite invoking fundamentally different mechanisms. The identification of neural signals that reflect some of the core computations underpinning decision formation offers new avenues for empirically testing and refining key model assumptions. Here, we highlight recent efforts to explore these avenues and, in so doing, consider the conceptual and methodological challenges that arise when seeking to infer decision computations from complex neural data.

## Highlights

Sequential sampling models have been widely embraced in contemporary decision neuroscience. The models come in many forms that, despite containing fundamentally different algorithmic elements, can make highly similar predictions for behaviour. Consequently, it can be difficult to definitively adjudicate between alternative models based solely on quantitative fits to behaviour.

The discovery of brain signals that reflect key neural computations underpinning decision-making is opening new avenues for empirically testing and refining model predictions.

Neurophysiological research is highlighting the multilayered neural architecture for implementing even the most elementary sensorimotor decisions. We do not yet know how many processing layers are required nor what distinct computations are performed at each layer.

## Decision-Making as a Core Component of Cognition

The term ‘decision-making’ often calls to mind scenarios such as voting in an election or selecting a course of study. Yet, even simply perceiving our sensory environment relies on a continuous stream of elementary judgments, known as ‘perceptual decisions’. In some cases, perceptual decisions can be as consequential as those requiring more abstract judgements (e.g., is the traffic light red or green?). In the highly complex and dynamic environment that we inhabit, making accurate and timely decisions is a considerable challenge for the brain, since the information it receives is almost always to some degree unreliable. Understanding how the brain overcomes the challenges associated with perceptual decision-making could also illuminate broader principles of computation that extend to a range of cognitive operations [1].

The theoretical foundations for modern research on perceptual decision-making were laid within mathematical psychology, with the development of ‘sequential sampling’ or **evidence accumulation** (see Glossary) models 2, 3, 4, 5, 6. These models have a long history of successfully accounting for choice behaviour in a range of contexts and, in addition, the core computations that they specify appear to be mirrored in certain components of neural activity in the rodent [7], monkey 8, 9, and human brain [10]. Consequently, recent years have witnessed a growth and confluence in research efforts to identify the computations through which perceptual decisions are formed, as well as to map, measure, and manipulate the neural structures and processes through which they are implemented, all anchored to the framework of sequential sampling. These continuing advances have given rise to an expanding repertoire of approaches combining neural and computational viewpoints [11]. In this review, we shine a spotlight on recent trends in using one such approach, where neural signals reflecting key aspects of bounded evidence accumulation are used to inform abstract decision models. We discuss the potential of this approach in providing strong grounds for model adjudication in cases where behavioural modelling alone falls short and, thus, for advancing important theoretical debates about decision computations. We also highlight the conceptual and methodological challenges involved.

## Abstract Decision Models and Challenges in Model Selection

Sequential sampling models were originally based on normative models for minimising the time taken to achieve a certain level of quality-control accuracy [12]. Sequential sampling models provide quantitatively accurate accounts of behaviour on a range of tasks, including perceptual detections and discriminations, lexical memory, response inhibition, and even social and value-based decisions (comprehensively reviewed in 13, 14). This powerful class of psychological process models can explain both random and systematic variations in performance. Furthermore, these models can decompose choice reaction times and accuracy into meaningful latent parameters, such as the strength of the evidence entering the decision process (‘drift rate’; i.e., the expectation of the evidence distribution being sampled) and the cumulative quantity required to trigger commitment (‘decision bound’). Ongoing research based on these behavioural models continues to fruitfully examine how decisions are shaped by factors such as speed pressure, value, prior knowledge, and distracting information, as well as how perceptual decisions are affected by brain disorders [14].

Many model variants exist because there are many alternative implementations of a decision process based on sequential sampling (Box 1). In many cases, competing model variants based on fundamentally different mechanisms can produce the same behavioural signature. This problem of model mimicry significantly hampers adjudication between competing accounts, and has given rise to several longstanding debates. To take an instructive example, there is ongoing disagreement about whether the criterion amount of evidence that we require to reach commitment can dynamically change during the course of a decision.

Box 1 **Sequential Sampling Models: Different Flavours for Different Research Objectives**

Over the years, several decision model variants have been developed based on the core principles of sequential sampling and bounded evidence accumulation. In standard, 1D diffusion models, for example, a sequence of samples from a Gaussian distribution representing noisy sensory evidence with, say, mean μ∆t (‘drift rate’) and variance Δt, is accumulated until the cumulant reaches an upper or lower bound. The drift rate scales with stimulus strength and the bounds are set to achieve a balance between speed and accuracy demands. The subject’s overall response time is modelled as a sum of the time it takes this diffusion process to reach the bound, and a ‘nondecision’ time accounting for additional delays associated with encoding, routing [100] and/or motor execution processes. In a popular, versatile version of this model, three of the parameters (the starting point, drift rate and nondecision time) are not fixed but rather can vary randomly from trial to trial, which provides significant flexibility to capture relatively fast or slow errors and specific RT distribution shapes [64].

Both simpler and more complex versions of this model have been developed, and the choice among these depends on research goals. In general, cognitive modelling is primarily concerned with forging abstract mathematical accounts of behaviour, the parameters of which serve as mechanistically interpretable metrics of task performance. Unlike neural or biophysical modelling, cognitive models do not generally strive to represent details of neurophysiological implementation [101]. Several reduced models have been developed to achieve this with computational ease, for example by excluding trial-to-trial variability parameters, where the relative speed of error responses is not critical [102], or by excluding the within-trial noise parameter (‘ballistic,’ racing accumulators 103, 104).

Toward the more complex end, the leaky competing accumulator model of Usher and McClelland [105] parameterises both the degree of competition between alternative accumulators and the leak of information within them, which provides one way to explain limited improvements in accuracy with longer viewing durations. Cortical microcircuit models have been developed that reproduce complex dynamical aspects of neural build-up patterns as well as decision behaviour 40, 106, and incorporate well-known motor control circuits, such as the basal ganglia [107]. An ongoing challenge is to establish a straightforward mapping between elements of these sometimes complex circuit models and the parameters of the more abstract models. Although cognitive and neural modelling have ostensibly distinct goals, there is valuable but underexploited territory at the interface between them, where models could capture key elements of neural implementation at distinct levels of the sensorimotor hierarchy as well as detailed behavioural trends.

Alt-text: Box 1

In the most widely subscribed models [13] (Box 1), although the bounds can be adjusted across different contexts to emphasise speed versus accuracy, in any given trial the bounds are assumed to be constant over time. Yet, ‘collapsing’ bounds (i.e., ones that decrease over time within a trial) provide an optimal policy according to normative theory under the common situation where evidence strength varies unpredictably across trials and is sometimes weak 15, 16, or where responses must be made within a strict deadline 15, 17. One of the main reasons why collapsing bounds have not been incorporated in the dominant models is because key behavioural consequences of doing so, such as decreased accuracy for trials with longer reaction times, can be also produced within a drift diffusion model with constant bounds, via an alternative mechanism involving trial-to-trial variability in drift rate [13] (Figure 1).

**Figure 1:**
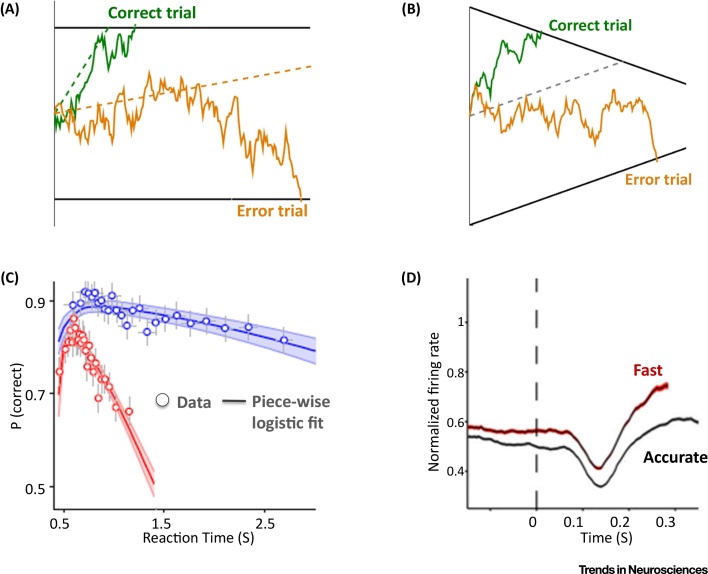
Alternative Mechanisms to Explain Why Choice Accuracy Reduces over Time within a Trial. (A) Schematic illustrating how drift rate variability with static bounds can produce slow errors. Solid lines indicate the path taken by a diffusion decision variable on each of two example single trials, one resulting in a correct response (green) and one resulting in an erroneous (orange) choice. Drift rate variability tends to produce response times that are longer, on average, for erroneous choices than for correct choices. Dotted lines mark the drift rate for each of those two trials. (B) Schematic illustrating how collapsing bounds without drift rate variability can alternatively produce slow errors. Again, two example single trials are shown, in this case arising from the same, fixed drift rate. (C) Conditional accuracy functions illustrating the decrease in accuracy as a function of response time (RT). Blue and red lines represent data from two different task conditions emphasising accuracy and speed, respectively. (D) Lateral intraparietal area (LIP) firing rate data highlighting that speed emphasis leads to an increase in the starting level of activity at trial onset and also an evidence-independent acceleration of signal build-up over time, reflecting a dynamic urgency component, the impact of which is equivalent to a collapsing bound (B). Panels C and D adapted from [25] and [91], respectively.

Establishing the relative prominence of these alternative mechanisms in choice behaviour has consequences beyond matters of preference in model-fitting approaches. These alternative accounts reflect fundamentally different algorithmic elements and, therefore, adjudicating between them has important implications for our understanding of normal and abnormal decision-making. For example, there has been an increasing application of sequential sampling models in studies seeking to better understand decision-making deficits observed in psychiatric populations 18, 19 or impairments associated with neurodegenerative disease 20, 21. If relatively slow response times were observed for error responses in a given clinical population (e.g., [21]), an explanation based on a faster bound collapse (e.g., due to a more impulsive decision policy or aversion to missed deadlines) would have different implications than one based on greater drift rate variability (e.g., due to fluctuations in attentional engagement, see below for further discussion), with respect to both explanatory accounts of the disorder and efforts to treat it. Similarly, an increasing trend in human neuroimaging research is to use decision model parameter estimates from behavioural data fits in statistical analyses to localise decision-relevant brain regions 11, 22. Here, again, the particular choice of model could have major consequences both for the particular areas identified and the interpretation of the role they might actually have in decision formation [23].

In behavioural model comparisons between two mechanisms that produce the same qualitative behavioural pattern, the outcome can greatly depend on the number and nature of the parameters used to implement those mechanisms. To take an example relevant to the above debate, Hawkins *et al.* [24] recently conducted formal model comparisons with several human and monkey data sets to adjudicate between collapsing bounds and drift rate variability. The comparisons were conducted using Bayes Information Criterion (BIC), which balances goodness of fit with **model parsimony**. The authors found that most data sets were better explained by a constant bound model with drift rate variability. Of note, however, in this comparison, collapsing bound models also included drift rate variability in addition to several parameters describing the collapse (nonlinear functions of time). As a result, the collapsing bound models were at a disadvantage, since BIC metrics penalise for complexity. In an attempt to address this, a second main comparison was made with a collapsing bound model contrived to have the same number of parameters as the constant bound model. Again, the data favoured constant bounds, but again questions remain, since the parameters that were omitted from the collapsing bound model were ones that account for qualitatively distinct and often significant aspects of behavioural data (e.g., fast errors and distribution shape). The simplest way to implement a collapsing bound (i.e., a linear function of time) was not considered. By contrast, a more recent study that did use such a linear implementation showed an improved BIC for a model that included collapsing bounds alongside drift rate variability [25].

## Neurally Informed Decision Models

Discrepancies such as the one discussed above highlight the difficulties that can arise when adjudicating between alternative models based on behavioural data alone. One approach to break such impasses is to additionally consider the ability of a model to capture key observable aspects of the biological implementation of the decision process 26, 27, 28, 29, 30, 31, 32. Advances in both animal and human neurophysiology have significantly broadened the possibilities for such an approach by identifying signals that exhibit key dynamical characteristics of bounded evidence accumulation. For example, in one line of work, single neurons in the monkey lateral intraparietal area (LIP) have been shown to exhibit strongly choice-predictive activity that builds at a rate proportional to physical evidence strength 33, 34, linearly grows in variance as more evidence is sampled over time [35], and reaches a stereotyped firing level immediately before the perceptual report [36]. More recently, human electrophysiology research has established that signatures of bounded evidence accumulation can also be traced in global, non-invasively recorded signals 25, 37, 38, 39 (Box 2). In parallel, empirically grounded, biophysically based models have been developed that describe plausible neural circuit configurations capable of implementing computations such as temporal integration (e.g., 40, 41). The ability to observe neural signals reflecting decision formation is not only relevant to the construction of such neural network models, but can also provide critical guidance in constructing, constraining, and adjudicating between abstract, cognitive process models. Returning to our example above, collapsing bounds and drift rate variability each make, in fact, specific predictions for neural signals relevant to decision formation, and many data already exist to examine such predictions.

Box 2 **Probing Decision-Related Neural Activity in Non-Invasive Recordings**

Significant advances in isolating decision signals from non-invasive human brain recordings open possibilities for translating the detailed characterisations of decision mechanisms wrought from nonhuman neurophysiology to the human brain in both health and disease. Moreover, global brain recording techniques, such as electro- and/or magneto-encephalography (EEG/MEG) and fMRI can complement intracranial investigations by offering a wider systems-level view of decision-related processes. However, a challenge is that non-invasive assays suffer from limited spatial or temporal resolution. In EEG/MEG, signals at the scalp reflect the sum of concurrently active components of neural activity. Several approaches have been used to disentangle the components specifically with a role in decision-making. One approach is to design paradigms that, by their nature, produce signals related to the core ingredients of a decision (e.g., sensory evidence, its accumulation over time, and emergent motor preparation) while minimising decision-irrelevant neural activity components. For example, decisions based on gradual changes in the intensity of flickering visual or auditory stimuli readily furnish sensory evidence signals through steady-state flicker-response amplitudes and eliminate irrelevant early sensory-evoked potentials normally evoked by sudden intensity transients [37]. This allows observation of decision formation dynamics relatively directly without imposing any constraints on the form they should take. The downside is that the approach works best for very elementary decisions.

Other approaches have used signal-analytic methods to extract decision-relevant signals during more complex tasks involving higher-order categorisations. For example, using a task requiring accumulation of orientation information varying stochastically over discrete sequential samples, sample-by-sample regression analyses can furnish distinct signal components related to decision-irrelevant sensory changes and relevant decision-update processes 108, 109. Another approach uses multivariate classification algorithms to derive functionally defined EEG components that, similar to the observers themselves, discriminate between blurred images of high-level objects, such as cars and faces [38]. Significant promise lies in combining the above paradigm-design and analytic approaches.

For the abovementioned non-invasive neurophysiology approaches, the ability to take measurements of dynamic decision signals at multiple hierarchical levels in the decision architecture has been demonstrated, yet the potential to use such measurements in neurally informed, or even neurally constrained, modelling is only beginning to be realised 25, 61. Joint neural–behavioural model fitting can also be done in a more data-driven manner, without necessarily singling out signals independently verified to reflect decision formation dynamics. This is best exemplified in neuroimaging research. Although limitations in temporal resolution preclude measurement of dynamics, brain-wide BOLD activations can be used as constraints in model fits [110] and have a vital role in identifying candidate decision-related brain structures for potential follow-up in intracranial investigations.

Alt-text: Box 2

Several recent neurophysiological studies in humans and monkeys have furnished evidence that decision bounds are, at least in certain contexts, adjusted dynamically during decision formation 25, 42, 43, 44, 45. For example, studying motion direction decisions, Hanks *et al.* [43] demonstrated that the spiking activity of neurons in area LIP, in addition to its dependence on direction and evidence strength, also exhibited an evidence-independent component of build-up for both choice alternatives, and this **urgency signal** rose more steeply under speed pressure (Figure 1D). By imposing a progressive reduction in the quantity of evidence needed to trigger commitment to any of the choice alternatives, urgency signals provide a neural mechanism for implementing the collapsing bounds proposed in mathematical models. In addition to this dynamic component, Hanks *et al*. also observed that LIP activity was elevated at the outset of the decision under speed pressure, consistent with an additional static component of the bound adjustment, and the findings of other human neuroimaging 46, 47, 48 and monkey [49] studies. Despite these starting point and time-dependent variations, LIP activity converged to a common level before the perceptual report. Based on these observations, a model that allowed for both static and dynamic adjustments to the decision bound was constructed. Crucially, the additional parameters describing these bound adjustments were not fit to the behavioural data but measured directly from neural activity, and the only parameters that were free to vary were ones that did not differ between the two speed pressure conditions. Nevertheless, the resultant model provided a compelling fit to the behavioural data, including the extent of the impact of speed pressure. Although it has been suggested that such urgency effects are peculiar to monkeys [13], and species differences of this nature likely do exist, consistent effects have recently been reported in human electrophysiological indices of motor preparation [25], suggesting that the effect is generalisable at least across primates. Alongside the growing number of empirical demonstrations of urgency and their increased incorporation into abstract models, new lines of research are seeking to identify plausible biophysical mechanisms for their generation. Neural network modelling studies have demonstrated the potential role of dynamic modulations of neural gain 50, 51, 52, in particular those mediated by neuromodulatory arousal systems [53], the dynamic activity of which can be empirically examined via changes to pupil diameter [25].

Drift rate variability is an undeniably convenient feature of abstract decision models for quantitative fitting of behaviour [54], but it is seldom scrutinised in terms of possible neurophysiological underpinnings. The most obvious candidate underlying cause is the random trial-to-trial fluctuation in the mean firing rates of neurons encoding **sensory evidence signals**. In the context of two-alternative decisions, such fluctuations would have to take the form of random biases towards one alternative or the other, rather than nonselective variations related to general arousal or task engagement, since drift rate is driven by differential evidence. Such fluctuations would also have to occur on the slow timescale of typical trial durations and, therefore, should give rise to significant and broad autocorrelation in evidence-encoding neurons. This has been examined in several areas, including monkey middle temporal visual area (MT) for motion decisions, where autocorrelation levels are, in fact, low and have short (on the order of <100 ms) timescales 55, 56, at least compared with higher brain areas [57]. This does not preclude variability in the weighting of such evidence signals as inputs to the accumulation process, and it is possible that broad fluctuations are more prominent in other sensory areas, other species, and/or other tasks. For example, during continuous monitoring for sensory targets occurring at highly unpredictable times, one could speculate that the absence of time constraints may minimise the influence of urgency signals, while the increased demands on sustained attention may yield trial-to-trial fluctuations in sensory evidence that impact the timing and probability of target detections [58].

In general, there are many different ways in which observations of decision-related neural signal dynamics can inform psychological process modelling and thereby help to converge on a computational account of the brain’s decision mechanisms 11, 30. The question of which is the most effective use of neural data depends on the nature of the data available, the paradigm used, and the particular mechanisms being examined. In the case of Hanks *et al.* [43], for instance, the particular set of stimulus conditions that was run enabled the time course of the urgency signal to be derived directly from the neural data and applied as a constraint in the model [59]. More generally, the correspondence between discrete measures of neural signal dynamics (e.g., onset time or rate of build-up of a decision signal) and model parameters (e.g., nondecision time or drift rate) may be more indirect, or lack the type of ‘one-to-one’ mapping that can provide definitive constraints for model parameters. In such cases, empirical neural dynamics can be compared with simulated model dynamics [30], which can be done in a couple of alternative ways.

One effective approach that is beginning to be used is to quantitatively fit a given model to both the neural signatures of decision formation and behavioural data combined in a single step [60]. This approach exploits a key benefit of **neurally informed modelling** in relying on the additional constraints brought by neural data to allow models to take on levels of complexity closer to the neural reality. Alternatively, in cases where behavioural data alone provide sufficient constraints for a reasonable fit, a ‘two-step’ approach can be taken, where behavioural fits are used to simulate dynamics for comparison with neural dynamics in a separate step. For example, in a recent study of rapid, value-biased sensorimotor decisions in humans [61], several candidate models invoking starting-point versus drift rate biases were first fit to behaviour. As found in most previous studies (e.g., 62, 63), a starting-point bias produced the better fit under the assumption of stationary (nontime-varying) drift rate. By contrast, a drift rate bias provided a better fit when drift rate was instead assumed to increase over time within a trial, to take account of the gradual nature of early sensory encoding processes when viewed on the timescale of very fast decisions. When evidence accumulation dynamics were simulated for all models, this value-biased, temporally increasing drift rate model made the unique prediction that neural signatures of decision formation should exhibit a ‘turnaround’ pattern on low-value sensory cues, where differential evidence is initially accumulated towards the wrong (but higher-value) alternative and is then dynamically rerouted towards the correct alternative. These very dynamics were observed in electrophysiological decision signals at both the level of motor preparation and motor-independent evidence accumulation. This study illustrates how qualitative model comparisons facilitated by electrophysiological signals tracing decision formation can bolster the outcomes of quantitative, behavioural model comparisons.

Neural signal analyses could similarly have a critical role in the application of models in research involving group comparisons. For example, consider the choice of ‘scaling parameter’, a parameter whose value is fixed, to anchor the model fit and to set the arbitrary scale on which all other parameters are measured (hence the name). A common choice in abstract decision models (e.g., the drift diffusion model, DDM) is to set within-trial noise to a fixed value [64]. However, is within-trial noise uniform across individuals or groups of individuals in reality? It is conceivable, for instance, that individuals with a certain clinical disorder would have greater within-trial noise compared with healthy individuals [65]. Differences such as this could in principle be observed directly through neural recordings, and help identify deficits among distinct mechanistic elements of the decision process.

An obvious caveat should be noted in relation to any of the above approaches: it must be taken into account how confident we are that the signals in question are indeed tracing the core neural computations that give rise to decisions [66]. Since many brain signals (e.g., sensory and motor) are likely to be correlated in some way with the observer’s choices, examining signal dynamics during the period of deliberation and establishing a temporal relationship between those dynamics and choice commitment (e.g., reaction time) is an essential step to avoid an erroneous attribution of function. Thus, as with fitting of behaviour alone, immediate-response paradigms that pinpoint the time of decision commitment provide critical constraints that enable more definitive model comparison 9, 36. In addition, it is important to take account of the fact that the roles of distinct brain areas and signals in decision-making are likely task dependent (see below and Box 3).

Box 3 **Causal Inference**

Much research effort in decision neuroscience has focused on recordings from area LIP, and this work has yielded insights into the computational mechanisms by which the brain accommodates speed–accuracy demands [43], prior biases [111], multiple alternatives [42], switching between alternate evidence dimensions [112], and other problems regularly faced by real decision-makers. As these insights have amassed, so also has the misconception that such findings imply that the central function of LIP is to accumulate evidence for decisions. This is of course misguided. LIP simply contains neurons the properties of which, characterised over decades of careful research into saccadic target selection 113, 114, make it possible to rigorously study certain transformations common to many decisions. To study these transformations, experimental conditions need to be carefully contrived so as to render LIP neurons informative in this context, for instance, by designing decision paradigms based on simple feature discriminations and on choices that are reported via saccades towards or away from targets placed within the receptive field of the recorded neuron. Moreover, these studies typically record from a subset of LIP neurons that exhibit sustained firing during delay periods before saccade execution, on the grounds that these neurons are likely best equipped to trace temporally extended decision processes. When one steps outside of these specific conditions, the choice-relevant dynamics observed in LIP can change substantially. For example, in the context of visual search, neural signatures of evidence accumulation are observed in the FEF 49, 75, whereas LIP activity has been linked more to the representation of salience as the core ‘evidence’ on which the search decision is based 115, 116. Even in the case of motion discrimination, LIP is only one of many areas carrying functionally similar evidence accumulation signals (e.g., [74]) In many of the decisions subjects face in their daily life, LIP, in fact, may not have a role at all. Even in the context of tasks involving saccadic choices, inactivation of LIP and rodent PPC has varying, task-dependent impact, but notably, has never been observed to be devastating to performance (e.g., 117, 118, 119, 120, 121). As stated at the outset of this line of work [33], the build-to-threshold dynamics in LIP do not in themselves suggest that decisions are formed in LIP, but rather that LIP can provide a window onto decision processes and onto the computations they implement, regardless of where the decision is initiated.

Alt-text: Box 3

## Accounting for a Multitiered Neural Architecture

**Figure 2:**
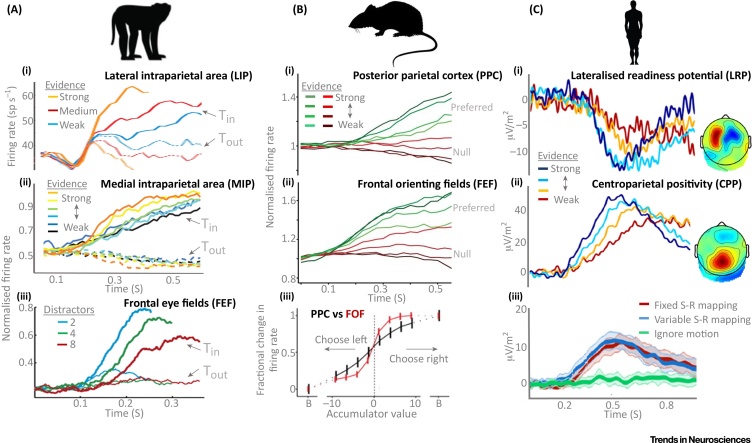
A Multiplicity of Decision Signals. (A) (i) When monkeys indicate motion direction discrimination decisions via saccade, neurons in the lateral intraparietal area (LIP) exhibit accumulation-to-bound dynamics that are highly sensitive to variations in sensory evidence. Here, LIP neuron firing rates increase more rapidly when coherent motion more strongly favours a saccade to a target located within the response field of the neuron (Tin). Although many intracranial recording studies of perceptual decision-making have targeted the LIP, similar neural decision signals have been observed in a variety of other regions of the monkey brain. (ii) When monkeys make reach movements to indicate their decisions, instead of saccades, reach-related neurons in the medial intraparietal area (MIP) exhibit similar accumulate-to-bound dynamics (unbroken traces). (iii) Movement neurons in frontal eye field (FEF) exhibit evidence accumulation dynamics during visual search decisions reported via saccade. Thin lines represent trials on which a distractor appeared within the response field of a neuron (Tout). (B) When rodents performed an auditory decision task, evidence accumulation dynamics are observed in (i) posterior parietal cortex (PPC) and (ii) frontal orienting fields (FOF). However, tuning curve analyses (iii) indicate that, while PPC provides a graded representation of incoming evidence, momentary FOF activity reflects the currently favoured alternative in a more categorical fashion. This pattern accords with the general observation from multisite recording studies that neural activity becomes progressively more closely linked to the observer’s action choices as one proceeds toward the motor end of the sensorimotor hierarchy. (C) When humans make motion discrimination decisions, highly similar accumulate-to-threshold signals are observed in non-invasive electrophysiological recordings. This work has uncovered two functionally distinct classes of decision signal: (i) when observers indicate their decisions via hand movement, contralateral motor preparation signals trace decision formation. These signals cease to trace decision formation if the stimulus-to-response mapping is withheld or when hand movements are not required. (ii) A centroparietal-positive (CPP) component in the event-related potential also traces evidence accumulation but does so irrespective of the sensory or motor requirements of the task. (iii) When participants withheld motion direction decision reports until the appearance of a response cue (1600 ms after stimulus onset), the CPP traced decision formation irrespective of whether the participant had foreknowledge of the stimulus-to-response mapping (fixed mapping) or not (variable mapping) and fell silent only when dot motion was rendered irrelevant to the task (ignore motion). Figures adapted from [36] (A.i), [74] (A.ii), [31] (A.iii), [7] (B.i-iii), [58] (C.i-ii), and [85] (C.iii).

Neurophysiological evidence from rodents, monkeys, and humans is increasingly highlighting the multilevel nature of the neural architecture of the brain for implementing even the most elementary decisions 7, 10, 67, 68 (Figure 2). If the purpose of a mathematical model is to simply account for the timing and accuracy of choice behaviour, representing explicitly each processing level is typically not necessary. However, if one wishes to develop a fuller systems-level picture of the neural decision process, and to pinpoint the origins of decision-making deficits, it is essential to understand how the distinct processing levels contribute to decision computations. In some cases, behavioural effects emanating from different processing levels can be disentangled through experimental design. For example, a recent behavioural study examined choice biases arising from differences in the energetic cost associated with reporting each alternative. The authors found that these choice biases did not originate at the motor level, as one might perhaps expect, but at an upstream level of decision formation that was independent from motor effectors [69].

In many cases, however, there are clear limits to the ability to localise effects among hierarchical processing levels using behavioural analysis alone. Several key parameters of sequential sampling models are likely subject to influences at multiple processing levels, and these influences often cannot be disentangled. For example, changes in the ‘nondecision time’ parameter (which accounts for delays due to processes not directly associated with evidence accumulation) could stem from altered delays at the outset of the decision process (e.g., sensory encoding) and/or at the end of it (e.g., motor execution). There is also ambiguity in the dependence of a parameter on changes at a single processing level versus in the transmission of information between levels; for example, drift rate is dependent not only on the strength and reliability of sensory representations themselves, but also on the weighting or reference values used in casting those representations as an input to the accumulation process (e.g., ‘drift criterion’ setting [64]).

Thus, there is much to be gained from examining decision-relevant neural dynamics at each of the key processing levels underpinning decision formation. A key challenge in this endeavour is that, even in the case of elementary sensorimotor decisions, we do not yet know how many levels of processing there truly are in the computational sense. Multiregion recordings have revealed that choice-selective signals are rapidly transmitted across many areas 70, 71 and, as one proceeds toward the motor end of the hierarchy, neural activity is progressively more closely associated with the subject’s action choice rather than the stimulus features 67, 72. However, beyond this general principle, the distinct role of each step of the pathway and its individual contribution to implementing the algorithm used by the brain to make a given decision are difficult to establish. In monkeys, for example, decision-related build-up activity with comparable latencies has been observed in LIP [73], medial intraparietal area [74] frontal eye field (FEF) 75, 76, prefrontal cortex 77, 78, superior colliculus [79], basal ganglia 80, 81, dorsal [82] and ventral premotor cortex [83], and primary motor cortex [44]. Not surprisingly, many research efforts have turned to identifying the distinct contributions that these areas make (Box 3, Figure 2).

Non-invasive human recording techniques can provide a more global view over several processing levels in tandem, although their lower resolution necessitates the use of paradigm designs and/or analysis methods that aim to disentangle their measurement (Box 2). Human electrophysiology studies have isolated two functionally distinct classes of decision signal reflecting accumulate-to-threshold dynamics: effector-selective signals that, similar to signals in areas such as LIP, represent the translation of sensory evidence into a specific motor plan 25, 39, 84, and a domain-general signal that builds with cumulative evidence regardless of whether responses are immediate, delayed, or not required at all, or of the sensory feature or modality being decided upon 37, 85 (Figure 2C). The latter supramodal, motor-independent signal, termed the ‘centroparietal positivity’ (CPP), was also found to precede evidence-selective motor preparation signals [58], further suggesting that it operates at a level of processing intermediating between sensory encoding and motor preparation.

This discovery not only builds on longstanding assertions that the brain must house abstract-level mechanisms to afford flexibility in mapping sensations to appropriate actions 86, 87, 88, 89, 90, but also refines this picture by suggesting that such intermediate processes can operate the way more dedicated circuits do; that is, by approximating an accumulation of sampled evidence towards a criterion or decision bound. The intracranial origins of this signal are as yet unknown. Given the similarity in bounded accumulation dynamics, it is tempting to link the CPP with activity in area LIP. However, EEG picks up neural activity globally and, since build-up activity for the selected alternative is mirrored by a roughly corresponding decrease in the activity of neurons coding for the unselected alternative, it would be expected that much or all of the choice-selective build-up activity of the LIP would be cancelled out at the level of the scalp. Interestingly, LIP neurons have been found to encode goal-relevant stimulus categories (e.g., motion direction) in an effector-independent fashion; however, it is not known whether these signals exhibit evidence accumulation dynamics [90]. More generally, much work remains to be done to understand the relationship between intracranial and extracranial signals exhibiting decision-predictive dynamics in different species [91] (Box 4). These questions notwithstanding, the identification of an abstract accumulation process in human brain recordings highlights the existence of an additional processing layer, the precise role of which in decision formation remains to be determined.

Box 4 **Bridging across Recording Modalities in Decision Neuroscience**

The neural bases of decision-making have been studied at a range of functional levels and scales, from single neurons, through neuronal microcircuits, to global activity measured in human electrophysiology and/or neuroimaging. With these expanding viewpoints comes the imperative to integrate findings across these levels. In part, this requires more general understanding of the biophysical translations between recording modalities. For example, in bridging from the neuronal circuit level to non-invasive electrophysiology, local field potential (LFP) activity and its relationship to multiunit spiking forms an important bridge to scalp EEG, which is thought to primarily reflect postsynaptic activity [122]. Such research has been increasingly undertaken recently at both the sensory level (e.g., [123]) and the level of emerging action plans (e.g., [124]). Studying the biophysical mechanisms by which extracellular LFPs translate to electric and/or magnetic signals at the scalp surface (e.g., [125]) and to BOLD activations (e.g., [126]) remains an active area of investigation.

Biophysically based computational modelling represents a complementary approach to integrating across levels of description while also specifying mechanisms of decision formation. For instance, spiking neuronal network models have successfully captured aspects of spiking dynamics and behavioural data during decision-making [40]. More recently, it was found that, through training, such recurrent neural networks can capture various idiosyncrasies found in neuronal population recordings, such as mixed, time-varying, and heterogeneous selectivity, across a variety of decision-making tasks 127, 128, 129. Such models reveal an additional layer of complexity of neural computation in decision-making, which may not be accomplished using simplified cognitive models.

Despite this progress, recurrent neural networks come with issues relating to stability and ease of interpretation with respect to decision algorithms of lower complexity. One means to bridge from spiking neuronal network models to simpler firing-rate, population-based models is through theoretical mean-field approximations 106, 130, but the application of this approach to heterogeneous networks is still in its infancy. Achieving a principled mapping of complex network models to lower-dimensional descriptions is vital to make linkages to the reduced cognitive models in widespread use in decision science [97], and has important implications for model-based analyses in neuroimaging, given the already prevalent reliance on neural mass models (e.g., dynamic causal modelling) to understand causal global brain dynamics [131], including in perceptual decision-making 132, 133.

Alt-text: Box 4

Although we may lack a complete picture of the essential computational layers for decision-making, studies that have recorded neural activity at multiple processing levels during the same task have already furnished insights that are beyond the reach of behavioural modelling alone. For example, recording from both MT and LIP during training on a motion direction discrimination task revealed that improvements in behavioural sensitivity with learning were attributable to changes in the motion-driven response of LIP neurons in the absence of any change in the evidence-encoding MT neurons, suggesting that learning changes the read-out but not the sensory representations themselves [92].

In certain instances, multiple levels of processing can be examined within a single brain area. For example, in the context of visual search decisions, salience-encoding visual FEF neurons provide the evidence that is accumulated by movement neurons, and these signals have also been used to directly constrain mathematical models 29, 31, 49. One such study examined the impact of speed and/or accuracy emphasis in visual search on processing at these distinct levels [49]. Despite the fact that behavioural data fits of a popular bounded accumulation model (linear ballistic accumulator, Box 1) indicated no difference in drift rate, speed pressure was found to enhance evidence encoding in visual FEF. Meanwhile, evidence accumulating movement neurons exhibited a complex pattern of adjustments that were not predicted by any pre-existing decision model, including increased activity levels at the time of saccade execution under greater speed pressure. The authors went on to construct a multilevel model that could accommodate this seemingly paradoxical finding by positing an additional leaky integration step carried out by brainstem neurons known to exhibit a threshold-crossing relationship with saccade execution and to receive direct projections from movement neurons of the FEF. This model provided as good a fit to the behavioural data as the standard model, while also capturing key qualitative features of the measured FEF activity, including increased build-up rate in the visual neurons under speed emphasis. This study highlights that, while abstract decision models can provide parsimonious accounts of choice behaviour, they may not necessarily capture all of the mechanistic steps that the brain performs and, therefore, are not always likely to correspond with neurophysiological dynamics observed at any one processing level. It also illustrates how models built from physiological knowledge of sensorimotor systems and their capabilities can have a pivotal role in facilitating the interpretation of decision-related neural activity patterns (Box 4).

Combining computational modelling with neural recordings probing multiple processing levels (e.g., sensory evidence encoding, motor-independent accumulation, motor preparation, and muscle activation) will be central to resolving a range of outstanding questions in the field. For example, thus far, much of the neurophysiological research on decision-making has focussed on activity in neural circuits situated close to the motor output end of the sensorimotor hierarchy. Therefore, we have a fairly refined picture of how key factors such as speed pressure, prior probability, and payoff information affect decision-making at this neurophysiological level, but a more limited picture on earlier processing stages. Of note, research on attention [93], feature expectation [94], and reward expectation 95, 96 has demonstrated the capacity of the brain to exert top-down influences on basic sensory representations. It remains unclear to what extent such modulations are used when adapting decision processes to account for contextual factors, and modelling studies rarely consider their potential computational benefits.

## Concluding Remarks

Sequential sampling models have provided a common, principled foundation to diverse investigations into decision-making. Behavioural fits of the models have long been used to furnish quantitative, mechanistically defined metrics to aid in understanding differences in how decisions are forged across stimulus conditions, task contexts, and clinical groups. However, the field has been grappling with several debates regarding key algorithmic elements of these models that are difficult to resolve based solely on quantitative fits to behavioural data. The ability to observe neural signal dynamics underpinning the decision process provides a means of guiding model development further. Recent studies demonstrate the unique insights that can be acquired by examining correspondences between abstract mathematical models and neural signals that have been independently verified to reflect elements of decision formation. It is now increasingly possible to construct models that are neurally constrained (e.g., quantitatively setting a time-varying stopping criterion based directly on neural measurements), neurally informed (e.g., including and fitting parameters for time-varying criterion settings based on qualitative patterns observed in the neural data), or at least neurally cognisant (e.g., including and fitting a time-varying criterion based on pre-existing neurophysiological evidence for its general role). With the ongoing development of techniques and paradigms for measuring decision-relevant neural processes, we can expect to see increasing adoption of such approaches that integrate neural evidence into computational accounts of decision-making (see Outstanding Questions). Adapting cognitive models to reflect the critical neural dynamics governing decision formation can also help substantially in establishing much needed linkages between the parameters and mechanisms of cognitive models and biophysically based neural circuit models, which are rarely brought into direct contact [97] (Box 4). The conceptual and methodological challenges examined in this review have implications that extend beyond research on perceptual decision-making because a trend toward integrating computational models and neural data is increasingly evident in many other research fields 98, 99.

Outstanding Questions Various factors are known to influence decision-making behaviour, among them: prior information, conflicting information, redundant information, energetic costs, spatial attention, perceptual learning, and value assignment. Processing of many of these factors is dysregulated in brain disorders. Do sequential sampling models provide accurate accounts of the essential neurocomputational adjustments through which these factors influence decision-making, and can neural signal analyses be used to determine whether that is the case? In addition to dominant criteria adjustments, are there modulations exerted at the sensory level that model fitting alone cannot detect?

The versatility of popular sequential sampling model variants is partly owed to the inclusion of certain parameters (e.g., variability in drift rate and starting point) that render the models flexible and enable them to account for different behavioural patterns. What predictions do these parameters make regarding neural activity, and how can these predictions be tested? Can neural signatures of such processes be identified?

Build-to-threshold decision signals have been observed in a variety of brain areas. What distinct computations do these signals and areas perform during decision formation?

What are the precise roles of abstract evidence accumulation signals in decision formation? What is the relationship between decision-related signals recorded non-invasively (e.g., in humans) and those observed in single-unit recordings (primarily in nonhuman primates and rodents)?

## Glossary

**Evidence accumulation**: according to sequential sampling models, accurate perceptual decisions can be achieved in the face of sensory noise by repeatedly sampling and integrating independent samples of evidence and withholding commitment until a predefined quantity has accrued in favour of one of the decision alternatives. There are multiple possible ways that this general process can be implemented both mathematically and neurophysiologically.
**Model parsimony**: mathematical decision models have traditionally been evaluated using statistical methods that balance the ability of a model to account for observed behaviour against its complexity. Evaluation methods that consider fits to neural as well as behavioural data are needed to facilitate the development of more detailed models that can account for the neural implementation of the decision process.
**Neural decision signal**: a neural signal that traces the process of decision formation. Typically, the term is used to distinguish neural computations that are tied solely to the choice outcome from sensory responses that exhibit trial-to-trial correlations with choice behaviour (see ‘Sensory Evidence Signal’ below). Here, we use the term primarily to refer to neural representation of accumulated evidence supporting decision formation. Single-unit and non-invasive electrophysiological recording studies have isolated signals exhibiting evidence accumulation dynamics that account for the timing and accuracy of the observer’s perceptual reports. The ability to directly observe and measure such signals opens new avenues for adjudicating between alternative decision models and developing new models that reflect the neural implementation of the decision process as well as its output.
**Neurally informed modelling**: the practice of basing model construction or constraining model parameters using qualitative and/or quantitative observations from empirical neural data. This approach contrasts with model-informed neuroscience approaches, in which an existing model is leveraged to furnish mechanistically defined behavioural metrics for correlation with neural data. For a comprehensive review of the distinct approaches to integrating mathematical and neurophysiological characterisations of decision-making, see 11, 66.
**Sensory evidence signal**: a signal that reflects the sensory input to a perceptual decision. Any stimulus will elicit a range of sensory signals, many of which may be irrelevant to the task at hand. The key distinguishing characteristics of a sensory evidence signal are that its momentary level should co-vary with a decision-relevant stimulus variable and its activity should predict choice behaviour in a stimulus-independent manner (also known as ‘choice probability’).
**Urgency signal**: an evidence-independent component of neural decision signal activity that expedites choice commitment. Such signals can be accommodated in mathematical models as a dynamic adjustment to the quantity of evidence required to trigger commitment (i.e., a collapsing decision bound). The recent identification of urgency signals that grow as a function of deliberation time challenges the dominant view in the mathematical modelling literature that, once adjusted, decision bounds remain fixed for the duration of a decision.
